# Efficacy of a paper-based interleukin-6 test strip combined with a spectrum-based optical reader for sequential monitoring and early recognition of respiratory failure in elderly pneumonia—a pilot study

**DOI:** 10.3389/fphar.2023.1166923

**Published:** 2023-05-05

**Authors:** Cheng-Han Chen, Yi-Chen Fu, Yi-Tzu Lee, Kai-Sheng Hsieh, Ching-Fen Shen, Chao-Min Cheng

**Affiliations:** ^1^ Department of Emergency Medicine, Taipei Veterans General Hospital, Taipei, Taiwan; ^2^ Institute of Biomedical Engineering, National Tsing Hua University, Hsinchu, Taiwan; ^3^ School of Medicine, National Yang Ming Chiao Tung University, Taipei, Taiwan; ^4^ Department of Pediatrics and Structural, Congenital Heart and Echocardiography Center, School of Medicine, China Medical University, Taichung, Taiwan; ^5^ Department of Pediatrics, National Cheng Kung University Hospital, College of Medicine, National Cheng Kung University, Tainan, Taiwan

**Keywords:** interleukin-6, point-of-care diagnosis, respiratory failure, community-acquired pneumonia, lateral flow immunoassay, elderly pneumonia

## Abstract

**Introduction:** Community-acquired pneumonia (CAP) is lethal in elderly individuals who are more vulnerable to respiratory failure and require more emergency ventilation support than younger individuals. Interleukin-6 (IL-6) plays a crucial role and has predictive value in CAP; high serum IL-6 concentrations in adults are associated with high respiratory failure and mortality rates. Early detection of IL-6 concentrations can facilitate the timely stratification of patients at risk of acute respiratory failure. However, conventional enzyme-linked immunosorbent assay (ELISA) IL-6 measurement is laborious and time-consuming.

**Methods:** The IL-6 rapid diagnostic system combined with a lateral flow immunoassay-based (LFA-based) IL-6 test strip and a spectrum-based optical reader is a novel tool developed for rapid and sequential bedside measurements of serum IL-6 concentrations. Here, we evaluated the correlation between the IL-6 rapid diagnostic system and the ELISA and the efficacy of the system in stratifying high-risk elderly patients with CAP. Thirty-six elderly patients (median age: 86.5 years; range: 65–97 years) with CAP were enrolled. CAP diagnosis was established based on the Infectious Diseases Society of America (IDSA) criteria. The severity of pneumonia was assessed using the CURB-65 score and Pneumonia Severity Index (PSI). IL-6 concentration was measured twice within 24 h of admission.

**Results:** The primary endpoint variable was respiratory failure requiring invasive mechanical or non-invasive ventilation support after admission. IL-6 rapid diagnostic readouts correlated with ELISA results (*p* < 0.0001) for 30 samples. Patients were predominantly male and bedridden (69.4%). Ten patients (27.7%) experienced respiratory failure during admission, and five (13.9%) died of pneumonia. Respiratory failure was associated with a higher mortality rate (*p* = 0.015). Decreased serum IL-6 concentration within 24 h after admission indicated a lower risk of developing respiratory failure in the later admission course (Receiver Operating Characteristic [ROC] curve = 0.696).

**Conclusion:** Sequential IL-6 measurements with the IL-6 rapid diagnostic system might be useful in early clinical risk assessment and severity stratification of elderly patients with pneumonia. This system is a potential point-of-care diagnostic device for sequential serum IL-6 measurements that can be applied in variable healthcare systems.

## 1 Introduction

Community-acquired pneumonia (CAP) remains the leading community-acquired infection threat resulting in a high hospitalization rate and mortality rate of 8.7%, associated with approximately three million annual deaths worldwide (Garau et al., 2008; World Health Organization, 2020). The incidence of CAP is higher in elderly individuals, accompanied by a higher associated mortality rate (10%–30% in severe cases) (Ray et al., 2006; Li et al., 2015; Schouten et al., 2019; Ferreira-Coimbra et al., 2020). Moreover, elderly patients are more vulnerable to severe complications, such as respiratory failure, requiring emergent ventilation support, and longer hospital stays (Kaier et al., 2019). Hence, early detection of pneumonia in elderly patients with an increased risk of developing respiratory failure is essential for delivering suitable treatments that may reduce the mortality rate (Kolditz et al., 2013).

Interleukin-6 (IL-6), a pro-inflammatory and pleiotropic cytokine secreted by stimulated monocytes and macrophages, mediates a broad range of immune responses after acute biological stress, such as trauma, infection, or inflammation (Andrijevic et al., 2014). This pleiotropic pro-inflammatory cytokine transmits signals, regulates immune cells, and generates a positive feedback loop. However, if dysregulated, the overproduction of IL-6 can ultimately induce a cytokine storm (Chen et al., 2022). In coronavirus disease 19 (COVID-19) patients, higher serum IL-6 concentrations imply hyperinflammatory responses that correlate with considerable disease severity and death. Indeed, IL-6 testing in COVID-19 patients has played a pivotal role in predicting poor prognoses and a higher risk of developing respiratory failure (Santa Cruz et al., 2021). In fact, elevated serum IL-6 concentrations may reflect disease severity and prognosis (Jekarl et al., 2013; Emami Ardestani and Zaerin, 2015; Song et al., 2019; Weidhase et al., 2019). In CAP patients, elevated serum IL-6 concentrations during hospitalization are associated with higher rates of respiratory failure and mortality (Andrijevic et al., 2014; Karhu et al., 2019).

Thus, it is crucial to measure serum IL-6 concentrations rapidly and repeatedly during treatment courses to facilitate the timely identification of either CAP or COVID-19 patients at risk of developing acute respiratory failure. However, the conventional method for quantifying IL-6, i.e., the enzyme-linked immunosorbent assay (ELISA), comprises various steps and requires several hours to generate results. These limitations impede the convenience of serum IL-6 measurements in current clinical practice (Schefold et al., 2008; Aydin, 2015).

A point-of-care (POC) diagnostic device may overcome these limitations. Hence, we propose the IL-6 rapid diagnostic system, comprising a lateral flow immunoassay (LFA)-based IL-6 test strip and a spectrum-based optical reader, as an alternative approach. This system has a reduced operating time of 1 h and exhibits a high correlation with conventional ELISA measurements in laboratory evaluation. Previous studies had found the IL-6 rapid diagnostic system to be well-suited for various clinical applications, including early diagnosis of respiratory failure risk in COVID-19 patients, differentiation of influenza severity in children, and early detection of acute wound infection (Lin et al., 2021; Pan et al., 2022; Wang et al., 2022).

However, the efficacy of rapid serum IL-6 measurements and early identification of elderly patients with pneumonia at-risk of respiratory failure remains unknown. Therefore, this study aims to assess the efficacy of the IL-6 rapid diagnosis system for the early identification of elderly patients with CAP who are prone to developing respiratory failure in the emergency department (ED) of a hospital in Taiwan. Such timely detection may improve patient prognosis and therapeutic outcomes.

## 2 Materials and methods

### 2.1 Patient selection

**FIGURE 1:**
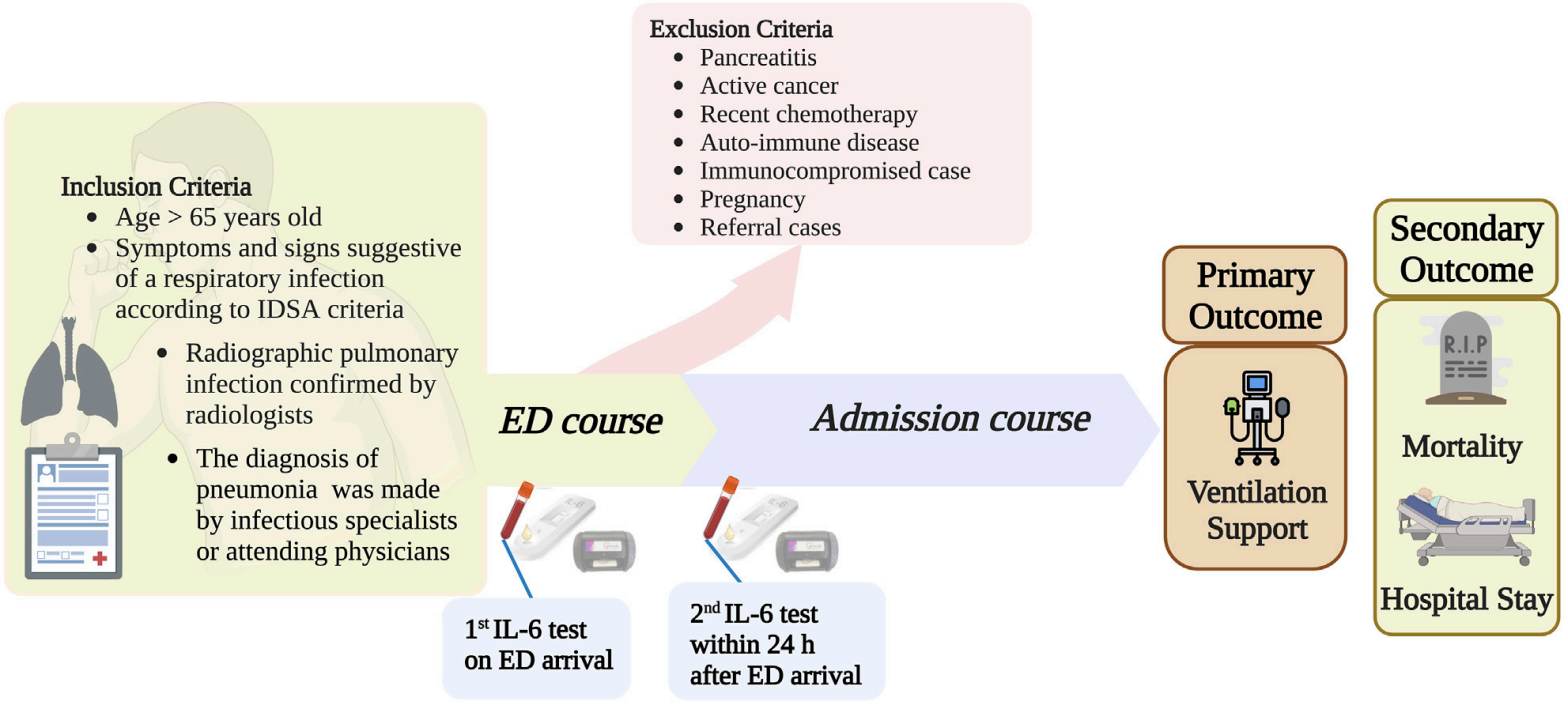
Length of hospital stay. Infectious Diseases Society of America; ED, Emergency Department. Figure created using Biorender.com.

This prospective, non-randomized, observational study was conducted from July 2021 to June 2022 at the ED of a medical center in Taipei Veterans General Hospital (TVGH), Taipei City, Taiwan. This study was conducted following the principles of the Declaration of Helsinki and was approved by the Institutional Review and Ethics Board (IRB) of TVGH (IRB: 2021-06-014AC). Written informed consent was obtained for all participants. For participants unable to provide informed consent due to cognitive impairment, caregivers consented on their behalf. The primary inclusion criteria were as follows: age >65 years; presentation to the ED with symptoms and indications of respiratory infection; established diagnosis of CAP according to the Infectious Diseases Society of America (IDSA) criteria (Metlay et al., 2019); chest X-ray (CXR) or thoracic computed tomography (CT) scan suggestive of CAP in the ED. The exclusion criteria were as follows: CXR or CT highly suggestive of lung cancer or other pulmonary metastatic lesions; ongoing chemotherapy or target therapy for cancer; immunocompromised; pancreatitis; transferred from another hospital, where symptoms developed for more than 5 days; non-compliant with protocol; or unwilling to participate in the study. Figure 1 demonstrates the study protocol.

Standard diagnostic procedures and treatments for CAP were performed for all patients screened for the trial. For those included in the study, complete blood counts with biochemistry panels were collected on the ED admission day. Standard CXR and/or CT were performed. Pneumonia Severity Index (PSI) and CURB-65 scores were assessed on the first day of hospitalization. On the first day, serum IL-6 concentrations were measured within 1 h of arrival at the emergency department (ED), and a second measurement was conducted within 24 h of ED admission on the following day. All surviving patients underwent follow-up until discharged. The primary outcome variable was respiratory failure requiring either invasive mechanical or non-invasive ventilation support after admission. The secondary outcome variables were mortality rate and length of hospital stay.

### 2.2 IL-6 rapid diagnostic system and ELISA for serum IL-6 concentration comparisons

All serum samples were preserved at −80°C until analysis. The serum IL-6 concentrations of 30 serum samples were measured using our IL-6 rapid diagnostic system and commercial ELISA kits (D6050; R&D Systems, Minneapolis, MN, United States) following the manufacturer’s instructions. The results were subsequently compared. The minimum detectable concentration was 3.1 pg/mL. The ELISA Sunrise Absorbance Microplate Reader was obtained from Tecan Sunrise™ (8,708; Tecan, Männedorf, Switzerland).

**FIGURE 2:**
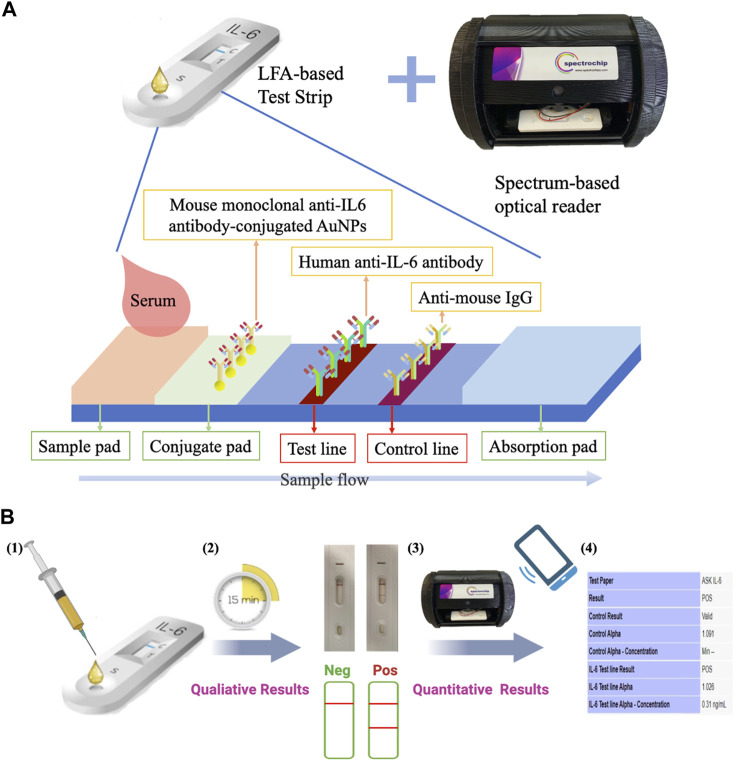
IL-6 rapid diagnostic system. **(A)** This system consists of a lateral flow immunoassay (LFA)-based test strip and a spectrum-based optical reader. The test strips consist of a sample pad, mouse monoclonal anti-IL-6 antibody conjugated gold nanoparticles (AuNPs) on the conjugate pad, human anti-IL6 antibody on the test line, and anti-mouse IgG on the control line. **(B)** Operation protocols: 1) 100 μL of the sample was placed onto the sample pad; 2) incubation for 15 min, allowing the IL-6 in the sample to bind to the AuNPs-conjugated monoclonal anti-IL-6 antibodies in the membrane as the sample diffuses through it to produce a visible colored line; 3) the color intensity is detected using the spectrum-based optical reader; 4) a readout is produced based on the designed software using the equation y = 0.0597*α*+1.0077, in which the calibration curve established based on the buffered system, a human albumin-based solvent (Lin et al., 2021).

The IL-6 rapid diagnostic system is a rapid and portable diagnostic system that was developed based on the principles of LFA-based IL-6 test strips in combination with a spectrum-based optical reader (Figure 2). Figure 2A illustrates the principles leveraged in the IL-6 test strips and optical reader, and Figure 2B shows the procedural protocol of the IL-6 rapid diagnostic system. For more details, the preparation of the LFA-based strips and spectrum-based optical reader have been described previously and presented in the Supplementary Material S1. (Lin et al., 2021; Wang et al., 2022).

### 2.3 Statistical analysis

Spearman’s rank correlation coefficient and Bland–Altman plot were employed to assess the correlation between the IL-6 rapid diagnostic system and ELISA results. The medians between different groups were comparatively analyzed using the Mann–Whitney U-test for non-normally distributed data. Thereafter, a Receiver Operating Characteristic (ROC) curve was generated to evaluate the relationship between the sequential change in serum IL-6 concentrations on ED arrival and the development of respiratory failure after admission. For all statistical results, *p* < 0.05 was considered significant. All statistical analyses were performed using IBM SPSS Statistics for MacOS (version 25.0; IBM Corp., Armonk, NY, United States) and GraphPad Prism (version 8.0; GraphPad Software Inc., San Diego, CA, United States).

## 3 Results

### 3.1 Comparison of IL-6 rapid diagnostic system and ELISA results in clinical samples

**FIGURE 3:**
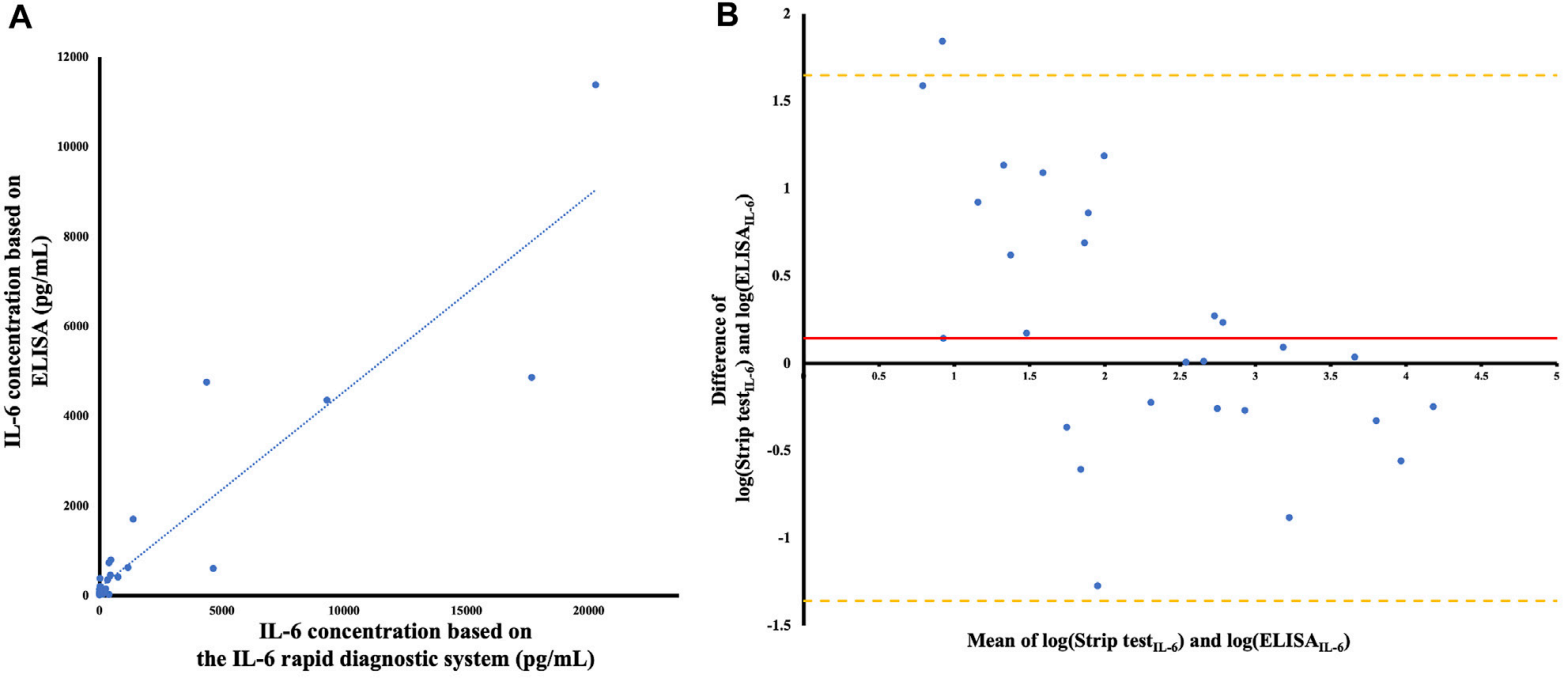
**(A)** Comparison of IL-6 concentrations with the IL-6 rapid diagnostic system and ELISA (*n* = 30). The blue line represents the linear regression relationship between the ELISA and the IL-6 rapid diagnostic system, with the equation y = 0.4369x + 180.88 (R^2^ = 0.8409). Spearman’s correlation demonstrated *Rho* = 0.7715 (confidence intervals 0.5619-0.8880, *p* < 0.0001). **(B)** Bland–Altman plot after logarithmically transforming the original data of the concentration. The red line demonstrates the mean difference between the ELISA and the IL-6 rapid diagnostic system, after logarithmical transformation, and the orange lines indicate the upper and lower Limits of Agreement (LOA) on the Bland–Altman plot.

We examined the IL-6 concentrations in 30 serum samples using the IL-6 rapid diagnostic system and ELISA. The correlation between concentration results was relevant and statistically significant (*Rho* = 0.7715, *p* < 0.0001; Figure 3A). Notably, the commercially available kits required samples to be diluted if the IL-6 concentration was >300 pg/mL (R&D Systems). In contrast, the rapid diagnostic system did not require any dilution prior to analysis.

To validate the correlation between these assays, we used the Bland–Altman analysis to assess any deviations from mean differences and estimate the interval of agreement (95%) between them. The Bland–Altman plot represents the comparison of the difference between the two methods with the measured average (Figure 3B).

After logarithmically transforming the original IL-6 concentration data based on the test strip and ELISA results, the mean difference between the assays was 0.8016, and their limits of agreement (±1.96 standard deviation) were 1.6477 and −1.3606, respectively. The width of the limits of agreement was 3.035, including most data points. A trend between the difference and mean was not observed; however, the difference between the methods was more variable when the mean was between 0.5 and 4.5.

### 3.2 Baseline characteristics, prognostic factors, and outcomes of all patients

A total of 36 patients were enrolled in the pilot study. The median age was 86.5 years, ranging from 65 to 97 years (Table 1). Most patients were male. Fourteen patients (38.9%) lived in nursing homes and 25 (69.4%) were bedridden and ADL-dependent (activities of daily living) (Kaur et al., 2018). No patients presented with respiratory failure upon ED arrival, however, ten (27.7%) experienced respiratory failure during the hospitalization course and received either invasive or non-invasive ventilation support. All patients with respiratory failure had a higher Charlson Comorbidity Index (*p* = 0.001), increased prevalence of dementia (*p* = 0.058), and chronic renal disease (*p* = 0.015). The prognostic scores (CURB-65 and PSI), duration of hospital stay, and mortality rate (*p* = 0.015) were higher in the respiratory failure group (*p* = 0.012).

**TABLE 1:** Baseline characteristics, prognostic factors, and outcomes of all patients.

	No (%) or Medium (IQR)			*P*
	Overall (*n* = 36)	Respiratory failure (*n* = 10)	Non-respiratory failure (*n* = 26)	
***Demographics***				
Female sex	9 (25%)	4 (40%)	5 (19.2%)	0.226
Age (years)	86.5 (81–92.75)	87.5 (83–95)	86.5 (80–92)	0.155
Smoker (Ex- or active)	4 (11.1%)	2 (20%)	2 (7.7%)	0.305
BMI (kg/m^2^)	20.79 (18.81–23.53)	20.51 (19.64–22.26)	20.84 (18.46–23.57)	0.838
***Baseline status***				
Nursing home residency	14 (38.9%)	5 (50%)	9 (34.6%)	0.462
Bedridden status	25 (69.4%)	9 (90%)	16 (61.5%)	0.127
***Comorbidities***				
Charlson Comorbidity Index	7 (5–8)	9 (8–9)	6 (4.5–7)	0.001
Dementia	15 (41.7%)	7 (70%)	8 (30.8%)	0.058
Past myocardial infarction	2 (11.1%)	2 (20%)	2 (7.7%)	0.305
Congestive heart failure	11 (30.6%)	5 (50%)	6 (23.1%)	0.224
Chronic pulmonary disease or asthma	6 (16.6%)	2 (20%)	4 (15.4%)	1.000
Diabetes mellitus	8 (22.2%)	4 (40%)	4 (15.4%)	0.179
Chronic renal disease	5 (13.9%)	4 (40%)	1 (3.8%)	0.015
***Prognostic score***				
CURB-65	3 (2–3)	3 (3–3)	3 (2–3)	0.145
Pneumonia Severity Index	155.5 (119.25–190.75)	194 (156–202)	138.5 (115–166.5)	0.012
***Outcome***				
Length of hospital stay (days)	16.5 (11.25–21.75)	22 (14–28)	15.5 (11–20)	0.166
Mortality	5 (13.9)	4 (40%)	1 (3.8%)	0.015

### 3.3 Vital signs and biomarkers upon ED arrival for the two severity groups

Table 2 presents the vital signs and biomarkers presented by elderly patients on arrival at the ED. The differences in vital signs between the severity groups and IL-6 concentration (*p* = 0.497) were not significant. The common prognostic biomarkers, such as white blood cell count, C-reactive protein (CRP), and procalcitonin (PCT) concentrations, did not differ between the groups.

**TABLE 2:** Vital signs and biomarkers upon arrival at the emergency department (ED) across different severity groups.

	No (%) or Medium (IQR)			*P*
	Overall (*n* = 36)	Respiratory Failure (*n* = 10)	Non-respiratory Failure (*n* = 26)	
**Vital Signs at ED visit**				
Heart rate	105 (76–118)	90 (73–132)	110 (80–117)	0.520
Respiratory rate	24 (20–26)	24 (23–30)	24 (20–26)	0.454
Temperature (°C)	37.3 (36.3–38.1)	37.2 (35.9–38.1)	37.3 (36.7–38.1)	0.715
Systolic blood pressure (mmHg)	142 (120–170)	148 (134–164)	134 (118–170)	0.958
**Biomarkers upon arrival**				
White blood cells (/µL)	12350 (7,725–16390)	12050 (7,120–16910)	12720 (8,950–15930)	0.958
C-reactive protein (mg/dL)	8.89 (2.77–12.36)	4.8 (1.12–11.36)	8.9 (3.94–12.01)	0.109
Procalcitonin (ng/mL)	0.21 (0.047–3.17)	0.10 (0.047–0.43)	0.45 (0.05–5.27)	0.434
Interleukin-6 (pg/mL)^a^	910.3 (54.8–4253.6)	441.32 (140.26–2922.71)	1215.99 (44.39–6340.00)	0.393

^a^ Measured using the interleukin-6, rapid diagnostic system.

### 3.4 Sequential change in the IL-6 concentrations between the different severity groups

**FIGURE 4:**
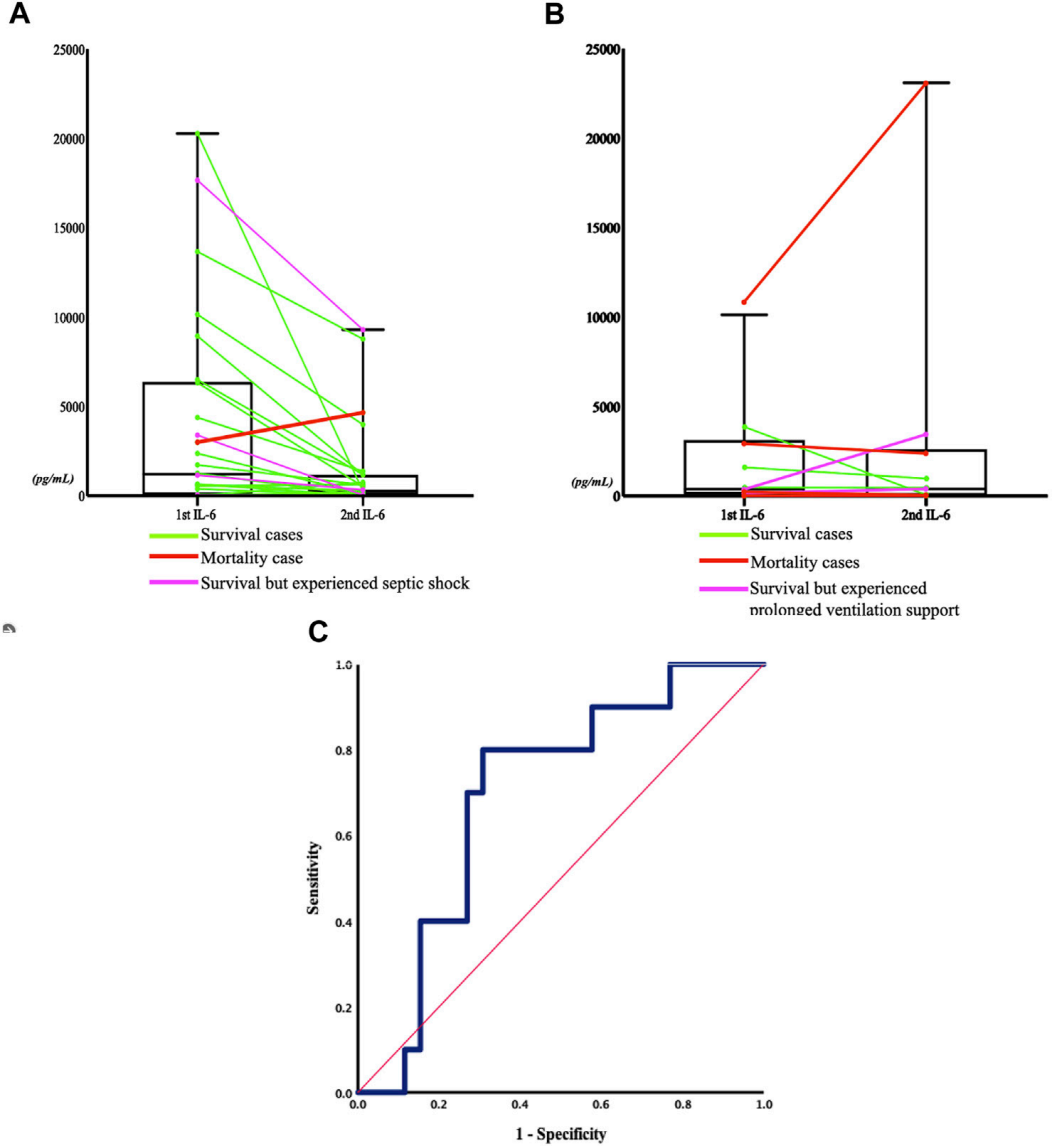
Sequential change in IL-6 concentrations between the two severity groups, and the receiver operating characteristic (ROC) curve of sequential IL-6 change and respiratory failure. **(A)** Non-respiratory failure group (p = 0.0676): the green line represents the patient who was discharged without experiencing septic shock nor requiring inotropic agents during admission, the pink line represents the patient who experienced septic shock, and the red line represents the patient who died within 5 days of admission. **(B)** Respiratory failure group (*p* = 0.8711): the green line represents the patient who was discharged, the pink line indicates the patient who experienced prolonged ventilation support (more than 21 days (Lone and Walsh, 2011)), and the red line indicates the patient who died within 5 days of admission. **(C)** The ROC curve (blue line) refers to the relationship between serum IL-6 concentration change after admission and the development of respiratory failure in the later hospitalization course. The area was 0.696 (95% confidential interval 0.515–0.877, *p* = 0.072). The Youden’s index of the ROC curve at **(A)** 43% change of the IL-6 concentration indicated that a decrease in IL-6 concentration below this threshold was associated with a higher rate of developing respiratory failure, with a sensitivity of 80% and a specificity of 69.2%. The red line represented the reference line.

IL-6 concentrations tended to decrease more in the non-respiratory failure group compared to the respiratory failure group (*p* = 0.071; Table 3). Figures 4A, B illustrate the sequential changes in IL-6 concentrations after ED arrival. The reduction in IL-6 concentrations in the non-respiratory failure group (*p* = 0.0676) was more prominent after admission than that in the respiratory failure group (*p* = 0.8711). Figure 4C illustrates the ROC curve referring to the relationship between changes in serum IL-6 concentrations after admission and the development of respiratory failure later in the hospitalization course (ROC area, 0.696; confidence interval 0.515–0.877, *p* = 0.072).

**TABLE 3:** Change in interleukin-6 concentration over time per severity group.

	Overall (*n* = 36)	Respiratory failure (*n* = 10)	Non-respiratory failure (*n* = 26)	*P*	AUROC^a^ with 95% confidence interval (CI)	*P*
	Medians with interquartile range					
Change of two sequential IL-6 (%)	−58%< (−93.64%–35.99%)	−1.85% (−39.35%–113%)	−69.49% (−98%–14%)	0.071	0.696 (0.515–0.877)	0.072
Time interval (h)	18 (12.5–24.5)	16 (8–25)	18.5 (15–24)	0.340		

^a^ The area under the receiver operating characteristic (ROC) curve.

## 4 Discussion

This observational study investigated the correlation between the IL-6 rapid diagnostic system and conventional IL-6 ELISA measurements, as well as the correlation between changes in serial serum IL-6 concentrations within 24 h of arrival at the ED and the possibility of developing respiratory failure in elderly patients with pneumonia after admission. The novelty of our study lies in our approach of monitoring the sequential change of interleukin-6 (IL-6) concentrations, rather than solely relying on threshold values, to predict adverse clinical outcomes such as respiratory failure in patients upon their arrival to the emergency department. This approach is supported by the findings of Lorenz Weidhase et al., who reported the clinical value of monitoring IL-6 as a predictor of antibiotic efficacy in 2019 (Weidhase et al., 2019). However, to the best of our knowledge, there has been little research into the sequential monitoring of IL-6 changes in the early stages of the disease to predict unfavorable outcomes. Therefore, we conducted a small-scale observational pilot study with a limited sample size to demonstrate the potential value of monitoring changes in serum IL-6 concentrations in the early recognition of disease severity and adverse outcomes. Our findings demonstrated the potential clinical significance of monitoring changes in IL-6 concentrations for early recognition of CAP patients at risk of respiratory failure, as evidenced by our ROC curve analysis, where the Youden’s index was found to be −43% (with a sensitivity of 80% and a specificity of 69.2%) when the percentage of IL-6 decrease was below this threshold.

Given the COVID-19 pandemic, the demand for easy-to-use, affordable tools for the early diagnosis of high respiratory failure risk or cytokine storm has increased. Compared to conventional ELISAs, which take approximately 4.5 h per run (R&D Systems, 2022), the total time required for our rapid test is less than 1 h. Therefore, the IL-6 rapid diagnostic system is a potentially applicable POC device for the timely and sequential measurements of serum IL-6 concentration. The IL-6 rapid diagnostic system demonstrated a strong correlation with conventional ELISA results in the buffered system, which utilized a human albumin-based solvent. The linear regression analysis showed an R^2^ value of 0.9507, and the limit of detection was determined to be 76.85 pg/mL (Lin et al., 2021). The maximum detectable concentration of serum IL-6 was approximately 25,000 pg/mL, as demonstrated in our study. Additionally, in previous clinical serum samples from children, the comparison between ELISA and the IL-6 rapid diagnostic system showed high relevance and statistical significance (Spearman’s *Rho* = 0.706, *p* < 0.001) (Lin et al., 2021). In this study, we also compared the measurements of the two methods using our clinical serum samples; the correlation was also significantly high (*Rho* = 0.7715, *p* < 0.0001), indicating that the IL-6 rapid diagnostic system can be an alternative methodology to conventional ELISA in serum IL-6 concentration measurement.

In this pilot clinical observational study, using the IL-6 rapid diagnosis system, we performed two successive examinations of serum IL-6 concentrations after ED presentation to predict respiratory failure which required invasive or non-invasive ventilation support in elderly patients with CAP. Although the number of patients was small (*n* = 36), the results suggest that a larger percentage of decreased serum IL-6 concentrations after admission indicated a lower risk of developing respiratory failure in late admission (ROC curve area = 0.696, *p* = 0.072).

CAP is a highly heterogeneous illness in terms of causative pathogens and diverse host responses (Metlay et al., 2019). The immune response during CAP can vary, as different microbes trigger unique inflammatory responses depending on the intrinsic properties of the pathogen. However, the causal agent is not the only determinant of its clinical outcome; interactions between the host immune response and pathogen clearance are essential. This balance between host immune interactions is regulated by complex interactions between immune cells and pro- and anti-inflammatory cytokines (Menéndez et al., 2012; Rendon et al., 2016; Mizgerd, 2018). Once the lungs are infected, the initial immune response is led by neutrophils, which release several granules and cytokines that can kill microbes and potentially cause tissue damage. Excessive or dysregulated inflammation activation results in exaggerated lung and systemic inflammation, which can cause severe complications (Fernandez-Botran et al., 2014). Elevated cytokine concentrations recruit more immune cells, such as macrophages, T cells, and neutrophils, to the infected area. If dysregulated, these immune responses can elicit various sequelae, such as endothelial destabilization, tissue damage, or multiple organ failure, which can result in a high mortality rate among infected patients (Ragab et al., 2020; Tabassum et al., 2021).

Among these inflammatory biomarkers, serum IL-6 concentrations increase rapidly after pathophysiological stress, compared to other common prognostic biomarkers such as procalcitonin (PCT) and C-reactive protein (CRP) (Karakioulaki and Stolz, 2019). Serum IL-6 concentrations increase immediately within 1 h of stress induction and peak at 3–6 h with a 15-h half-life (Gebhard et al., 2000; Wirtz et al., 2000; Shimazui et al., 2017; Kuribayashi, 2018). On the contrary, PCT increases within 4 h after infection and peaks at 6 h (Rowland et al., 2015), and CRP increases within 12–24 h and peaks 20–72 h after pathophysiological stress. (Mooiweer et al., 2011; Rowland et al., 2015; Hahn et al., 2018). The half-life of IL-6 in serum is relatively short compared to that of PCT (approximately 24 h) and CRP (19 h) (Biffl et al., 1996; Pepys and Hirschfield, 2003; Samsudin and Vasikaran, 2017; Chen et al., 2022).

Following the COVID-19 outbreak repeated IL-6 measurements in patients with COVID-19 and pneumonia were proposed as significantly lower serum IL-6 concentrations after admission were associated with a higher recovery rate (Liu et al., 2020). Moreover, several studies have reported that IL-6 may be a useful predictor of treatment failure and mortality (Heinrich et al., 1990; Biffl et al., 1996; Karakioulaki and Stolz, 2019). Indeed, the mortality was highest in patients with higher IL-6 concentrations (Kellum et al., 2007). Regarding CAP cases, repeated evaluation of patients with pneumonia following hospital admission is essential as clinical deterioration and mortality rates are the highest within 24–72 h of hospitalization (Kolditz et al., 2013). Compared to CRP and PCT, serum IL-6 concentrations provide a better predictor for monitoring antibiotic treatment in septic patients, in which a reduced serum IL-6 concentration indicates a better response to treatment (Weidhase et al., 2019). In contrast, consistently high serum IL-6 concentrations suggest continuous production of IL-6 and may indicate unresolved insults, such as trauma or infection.

Meanwhile, IL-6 is designated as a “gerontologist’s cytokine” because it is associated with various age-related chronic illnesses, the transition from innate to acquired immunity, and metabolic control (Ershler, 1993; Rea et al., 2018). Immunosenescence, a process of immune dysfunction that occurs with age, provokes a reduction of naive lymphocytes, accumulation of memory and effector lymphocytes, fabrication of defective antibodies or autoantibodies, and chronic low-grade inflammation status. Thus, advanced age with chronic inflammation is associated with substantially increased IL-6 concentrations (Ershier et al., 1994; Dobbs et al., 1999; Puzianowska-Kuźnicka et al., 2016; Lin et al., 2018). Hence, when managing sepsis in elderly patients, it might be more critical to monitor the sequential changes in serum IL-6 concentrations rather than measuring them once above a designated, detectable threshold.

In our study, decreased sequential serum IL-6 concentrations were found to potentially indicate better outcomes among elderly patients in both groups. Table 4 demonstrates the changes in IL-6 concentrations in the two groups. The green line represents the patients who were discharged following successful treatment for pneumonia, without complications such as septic shock and the respiratory failure group where the patients had removed their ventilation support within 14 days. The red line represents the patients who died of pneumonia during the later admission courses. Notably, two patients whose serum IL-6 concentrations increased after admission died within 10 days of admission. The pink line indicates patients who required prolonged ventilation support (of more than 21 days (Lone and Walsh, 2011)) or had suffered septic shock and required inotropic agents support, whose serum IL-6 concentrations were persistent or increased. In summary, increased or persistent serum IL-6 concentrations may be indicative of a poor response to treatment and poor outcome (Biffl et al., 1996; Kellum et al., 2007).

**TABLE 4:** Details of sequential serum IL-6 changes and patient outcomes.

	Respiratory failure	Non-respiratory failure
Green Line	4 patients who were weaned off of ventilation support within 14 days were discharged	21 patients without septic shock nor required inotropic agents were discharged
Pink Line	2 patients received prolonged ventilation support (more than 21, 26, and 35 days)	4 patients who required inotropic agents during hospitalization were discharged
Red Line	4 patients died of complications with septic shock, wherein 2 died within 10 days after admission (5 and 10 days)	1 patient died of septic shock on the fifth day after admission

This study has several limitations. First, as a pilot study to evaluate the correlation between sequential IL-6 concentrations and respiratory failure, the number of patients was small, with some of the results not reaching statistical significance. Second, as this study is a pilot study, the sample size may be limited in conducting sub-analyses for outcomes among different age ranges, sex, or other factors. Additionally, pneumonia is caused by various pathogens, each with its own intrinsic properties that can potentially impact clinical outcomes. Therefore, including a larger sample size in subsequent analyses would be beneficial in reducing or conducting sub-analyses to account for the effect of pathogen heterogeneity and further validate our hypothesis. Third, we only assessed the initial two serum IL-6 concentrations after ED admission, while more frequent measurements, such as three or four consecutive evaluations, can provide more information for facilitating treatment and positive patient outcomes.

**FIGURE 5:**
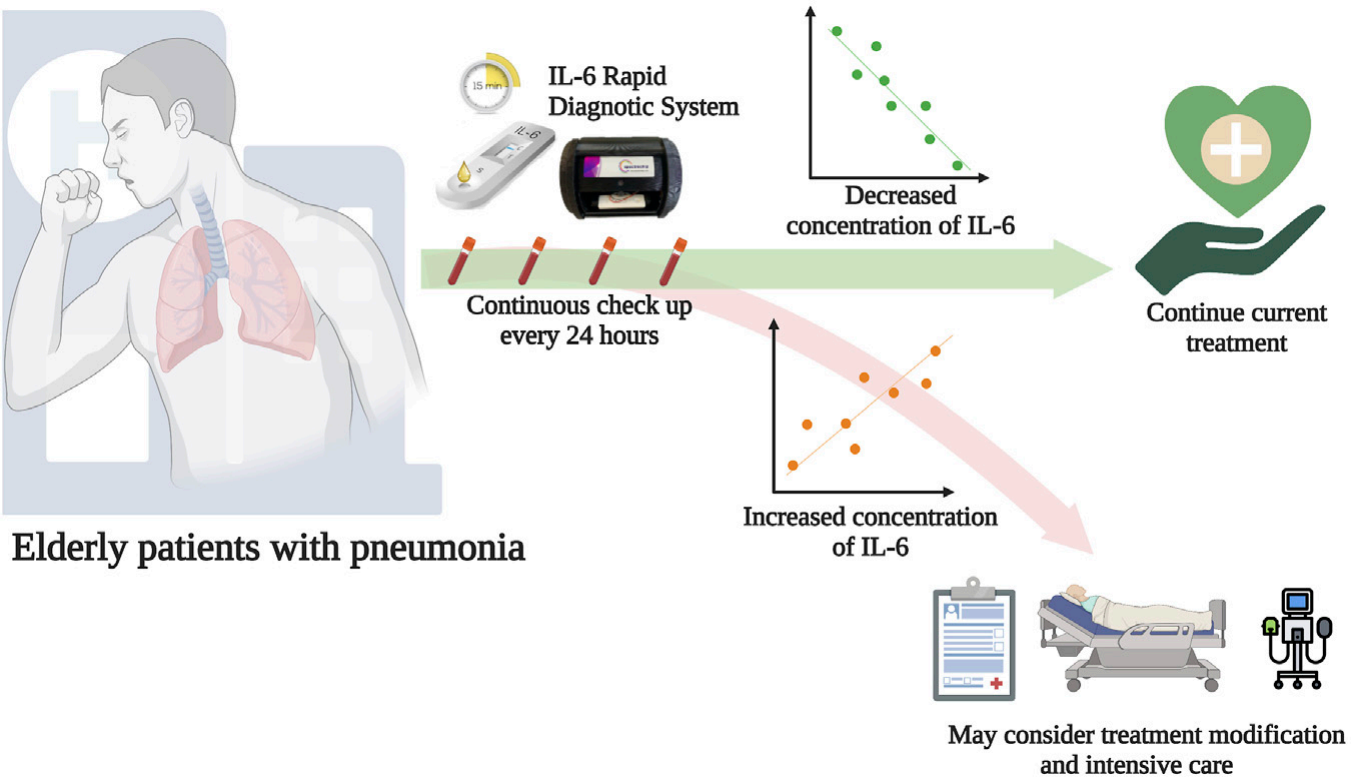
Potential application of the IL-6 rapid diagnostic system. If elderly patients who have been diagnosed with pneumonia visit the emergency department, sequential measurements of serum IL-6 concentrations should be performed. 1) If the concentration of serum IL-6 decreases, healthcare providers can maintain the current treatment; 2) If the concentration of serum IL-6 increases, healthcare providers should review the patient’s current condition, consider treatment modification, and provide intensive care monitoring. Figure created using Biorender.com.

In conclusion, we have described a novel methodology to execute sequential IL-6 measurement rapidly and easily using a system that combines an IL-6 LFA test strip and a spectrum-based optical reader. The results of the IL-6 rapid diagnostic system were highly consistent with the results of the conventional ELISA examination. Meanwhile, we demonstrated that sequential serum IL-6 concentrations measurement in elderly patients with pneumonia is more important than simply determining whether the serum IL-6 concentration is above a certain threshold value at a single time point. While the ELISA method is considered the gold standard for IL-6 measurement and has a lower LOD (3.1 pg/mL vs. 76.85 pg/mL) compared to the IL-6 rapid diagnostic system, its complexity hinders its practicality (Lin et al., 2021). The IL-6 rapid diagnostic system, as a novel medical device, has the potential to serve as an ideal alternative with comparable results. The IL-6 rapid diagnostic system offers several advantages, including a significantly shorter turnaround time, with results available in just 30 min, compared to the approximately 4.5 h required for the ELISA test. Additionally, our system allows for the analysis of a single sample at any time or the simultaneous analysis of multiple samples on different test strips (15 min per test), followed by an examination in an optical spectrum reader (5 min per test) one by one. Hence, we propose the use of a point-of-care IL-6 rapid diagnostic system, which has a non-inferior performance to the ELISA test, as a tool for facilitating early detection of patients at risk of respiratory failure or disease deterioration. This POC system can provide an alternative method for rapid and repeated IL-6 measurement, allowing for the sequential measurement of bedside treatment response (Figure 5).

## Data Availability

The raw data supporting the conclusion of this article will be made available by the authors, without undue reservation.
