# Corrigendum: Efficacy of a paper-based interleukin-6 test strip combined with a spectrum-based optical reader for sequential monitoring and early recognition of respiratory failure in elderly pneumonia-a pilot study

**DOI:** 10.3389/fphar.2023.1250358

**Published:** 2023-11-13

**Authors:** Cheng-Han Chen, Yi-Chen Fu, Yi-Tzu Lee, Kai-Sheng Hsieh, Ching-Fen Shen, Chao-Min Cheng

**Affiliations:** ^1^ Department of Emergency Medicine, Taipei Veterans General Hospital, Taipei, Taiwan; ^2^ Institute of Biomedical Engineering, National Tsing Hua University, Hsinchu, Taiwan; ^3^ School of Medicine, National Yang Ming Chiao Tung University, Taipei, Taiwan; ^4^ Department of Pediatrics and Structural, Congenital Heart and Echocardiography Center, School of Medicine, China Medical University, Taichung, Taiwan; ^5^ Department of Pediatrics, National Cheng Kung University Hospital, College of Medicine, National Cheng Kung University, Tainan, Taiwan

**Keywords:** interleukin-6, point-of-care diagnosis, respiratory failure, community-acquired pneumonia, lateral flow immunoassay, elderly pneumonia

In the published article, there was an error in the legend for (Figure 4) as published. [**(A)** Non-respiratory failure group (*p* = 0.0676): the green line represents the patient who was discharged without experiencing septic shock but required inotropic agents during admission,]. The corrected legend appears below.

[**(A)** Non-respiratory failure group (*p* = 0.0676): the green line represents the patient who was discharged without experiencing septic shock nor requiring inotropic agents during admission,]

**FIGURE 4:**
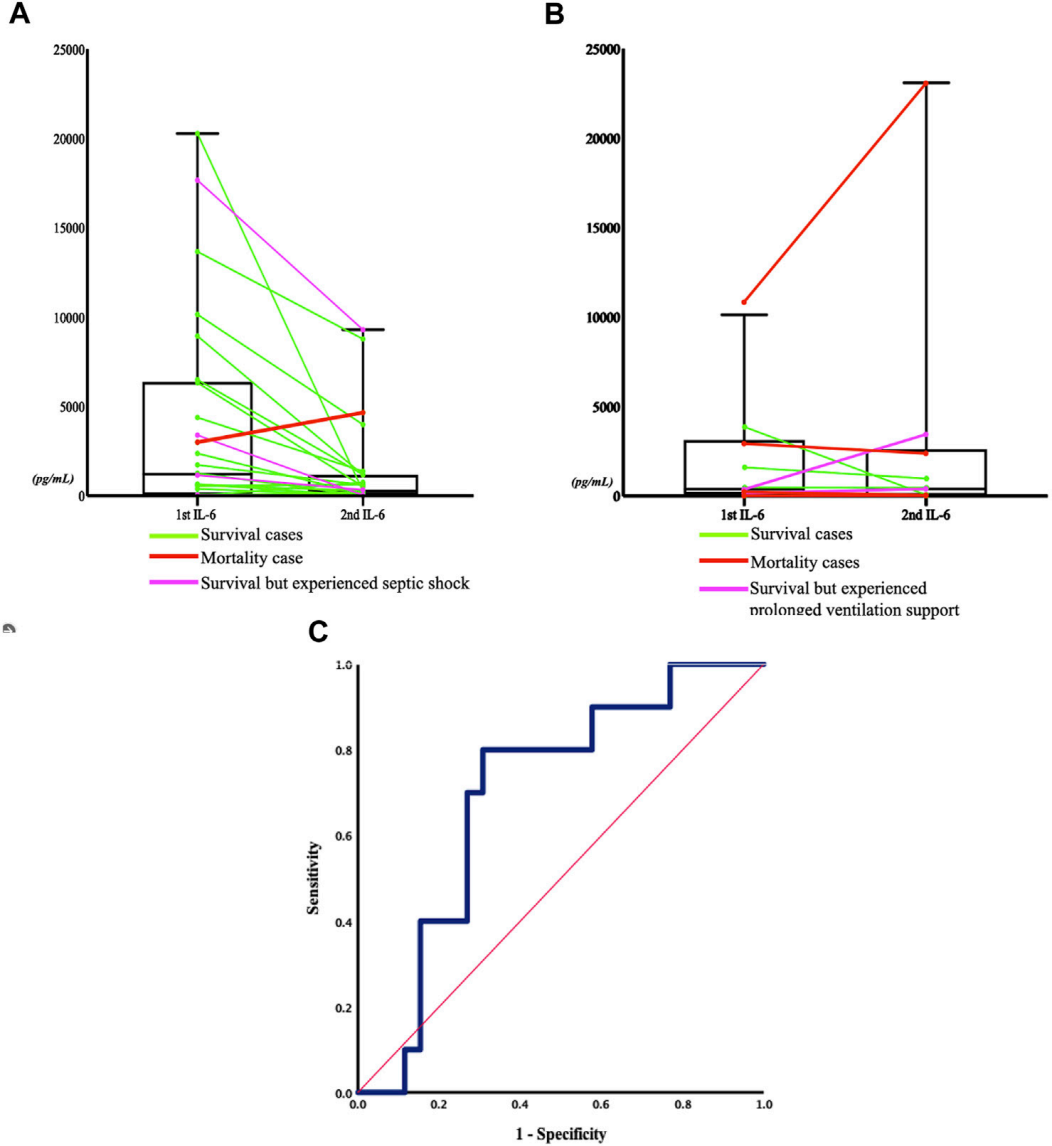
Sequential change in IL-6 concentrations between the two severity groups, and the receiver operating characteristic (ROC) curve of sequential IL-6 change and respiratory failure. **(A)** Non respiratory failure group (P = 0.0676): the green line represents the patient who was discharged without experiencing septic shock nor requiring inotropic agents during admission, the pink line represents the patient who experienced septic shock, and the red line represents the patient who died within 5 days of admission. **(B)** Respiratory failure group (P = 0.8711): the green line represents the patient who was discharged, the pink line indicates the patient who experienced prolonged ventilation support (more than 21 days (Lone and Walsh, 2011)), and the red line indicates the patient who died within 5 days of admission. **(C)** The ROC curve (blue line) refers to the relationship between serum IL-6 concentration change after admission and the development of respiratory failure in the later hospitalization course. The area was 0.696 (95% confidential interval 0.515–0.877, P = 0.072). The Youden's index of the ROC curve at a -43% change of the IL-6 concentration indicated that a decrease in IL-6 concentration below this threshold was associated with a higher rate of developing respiratory failure, with a sensitivity of 80% and a specificity of 69.2%. The red line represented the reference line.

In the published article, there was an error in (**Table 4**) as published.

The Section [21 patients (without septic shock) who required inotropic agents were discharged,] is incorrect. The corrected section is as follows.

[21 patients without septic shock nor required inotropic agents were discharged,]

The authors apologize for this error and state that this does not change the scientific conclusions of the article in any way. The original article has been updated.

