# Supplementary material for: Efficacy of a paper-based interleukin-6 test strip combined with a spectrum-based optical reader for sequential monitoring and early recognition of respiratory failure in elderly pneumonia—a pilot study
Source: Front Pharmacol. 2023 May 5;14:1166923. doi: 10.3389/fphar.2023.1166923 (PMC10196015; doi:10.3389/fphar.2023.1166923)
Supplement: Supplementary file 1 [file DataSheet1.pdf]

Supplementary Materials

Efficacy of a Paper-Based Interleukin-6 Test Strip Combined with a Spectrum-Based Optical Reader for Sequential Monitoring and Early Recognition of Respiratory Failure in Elderly Pneumonia—A Pilot Study

Preparation of the LFA-based strips combined with a spectrum-based reader

To rapidly detect human IL-6, an LFA-based test strip was produced in cooperation with Taiwan Hygeia Touch Incorporation, with the integration of colloidal gold nanoparticle (AuNP)-conjugated anti-IL-6 antibody (ARG21446; Arigo Biolaboratories Corp., Hsinchu, Taiwan).

The LFA-based IL-6 strips were sprayed with capture reagents to create vertical lines of human anti-IL-6 antibodies (ARG21444; Arigo Biolaboratories Corp). An optimization examination was performed to determine the most suitable formula for IL-6 concentration and AuNP size for conjugation. Optimization and cross-reactivity tests with various endogenous substances (bilirubin, hemoglobin, intralipid, rheumatoid factor, IL-1α, IL-1β, IL-2, IL-3, IL-4, IL-8, and IL-9) have been described previously (Wang et al., 2022; Lin et al., 2021).

The LFA principles:

The IL-6 test strip structure consists of the sample layer, nitrocellulose membrane, modification layer, and water-absorbing layer. The surface test strip structure consists of the sample pad, conjugated pad (coating with mouse monoclonal anti-IL6 antibody conjugated AuNPs), T line (Human anti-IL-6 antibody), C line (Anti-mouse IgG), and an absorption pad. As the sample fluid is dripped on the sample pad, it becomes pulled by force generated through capillary action, driving the sample fluid flow to the absorption pad. If the test line turns red, the IL-6 concentration of the targeted sample is above the threshold value set by the test strip or a positive result. That is when the concentration of IL-6 in the targeted sample exceeds the threshold value established by the test strip, the detection and control lines will turn red and show two lines (a positive result). Or, if the concentration of IL-6 is below the threshold, or no IL-6, in targeted samples, only the control line on the test strip will turn red (a negative result).

To gain quantity results from IL-6 test strips, we analyzed the spectral reflectance of anti-IL-6 antibodies coupled with various concentrations of serum IL-6 through the Tecan Sunrise™ Absorbance Microplate Reader (8708 Männedorf, Switzerland: Tecan). Previous studies showed that the difference in the absorption spectrum among various IL-6 concentrations is between 470 nm to 550 nm (Lin et al., 2021). The results can be attributed to the effect of light absorption of nanogold particles (nano-AuNPs).

To make the diagnostic system portable, the "IL-6 rapid diagnostic system" integrates an optical spectrum reader (Taiwan FDA: MD(I)-008090 and US FDA: 3017810861), which was made in

cooperation with Taiwan SpectroChip Inc. Figure S1 provides a visual representation of the optical spectrum reader. The optical spectrum reader exhibits a compact and lightweight design, boasting precise dimensions of 155 mm in length, 105 mm in width, and 125 mm in height. Its weight is estimated to be approximately 1.5 kg, rendering it an efficient and portable device for optical spectrum analysis. The optical reader contains a cassette for the stripe to assess the spectral reflectance on the strip, reading the IL-6 concentration more quickly. This optical reader records continuous spectral reflectance values of the test line (IL-6 examination line). Following a previous study, the ELISA reader reads the wavelengths at 540 nm, 470 nm, and 650 nm as a reference (Hung et al., 2021). With the optical reader, the LOD of the IL-6 rapid diagnostic system is ameliorated from 40 ng/mL in IL-6 strips only to 76.85 pg/mL (Lin et al., 2021). AuNPs do not absorb 650 nm light; thus, the various shade of red lines yields minimal change at 650 nm, which equals 1. The α value is computed utilizing the proportion of the spectra between the minimum (430–600 nm) and reference (650 nm) wavelengths, as represented by the following equation:

α = Reflectance (650 nm)/Reflectance (the minimum value in the range of 430–600 nm).

A higher α value represents a more prominent reflection, referring to more AuNPs antibody-conjugated IL-6 complex on the T line, suggesting a higher IL-6 concentration (Wang et al., 2022; Lin et al., 2021; Pan et al., 2022). However, the calibration curve of the IL-6 rapid diagnostic system established from the buffered system, a human albumin-based solvent, and the efficacy of clinical application remained unknown.

The current protocol for the IL-6 rapid diagnostic system is as below (Figure 2B in the main manuscript).

1) Serum (100 μL) is dropped into the sample application pad on the test strip, and pre-treated with an AuNP-conjugated monoclonal antibody specific for IL-6.
2) After waiting 15 min, colorimetric intensity is assessed with a spectrum-based optical reader.
3) Qualitative readouts are calculated based on a lot-specific calibration curve.
4) The readout produced by a designed software based on an *estimated IL-6 concentration = 0.0597α + 1.0077*, calibration curve established based on the buffered system, a human albumin-based solvent (Lin et al., 2021).

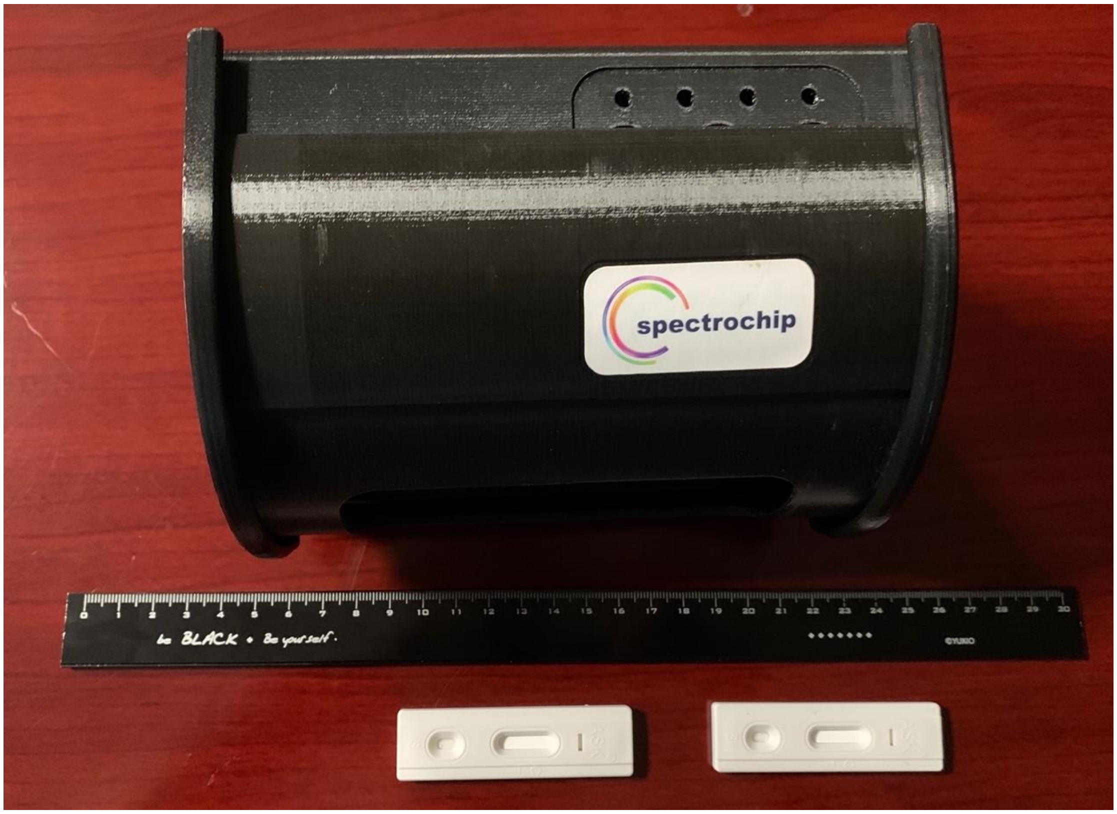

Figure S1. The optical spectrum reader. This figure presents a visual representation of the optical spectrum reader, highlighting its compact and lightweight design. The precise dimensions of 155 mm in length, 105 mm in width, and 125 mm in height are depicted, with a ruler provided for scale reference. Two white cassettes, representing the IL-6 test strips, are shown below the ruler. The estimated weight of approximately 1.5 kg further emphasizes the efficiency and portability of this device for optical spectrum analysis.

Table S1. The IL-6 concentrations among males and females in different groups.

		**IL-6 concentration on arrival**	**IL-6 concentration within 24 hours**	**The change in IL-6 concentration**	**Comparison within the group**	**Inter-group sex differences within the same sex.**	
Respiratory Failure	Male	303.89 (73.03-2158.38)	220.94 (32.21-419.65)	48.49% (-51.17%-139.06%)	P = 0.9143 (C.I. -177.4 to 870.1)	Male (Respiratory versus Non-respiratory)	P=0.2887 (C.I -194.7 to 36.45)
	Female	975.71 (177.56-2259.51)	1670.94 (484.09-2902.65)	-9.39% (-29.07%-433.18%)			
Non-respiratory Failure	Male	85.71 (25.31-4388.40)	29.06 (0-263.63)	-98.70% (1-00%- -68.57%)	P = 0.6705 (C.I. "-110.4 to 42.56")	Female (Respiratory versus Non-respiratory)	P=0.111 (C.I -961.9 to 275.2")
	Female	630 (391.55-658.29)	33.24 (24.95-105.53)	-91.51% (-95.57- -56.14)			

Table S1 presents the IL-6 concentrations among males and females in different groups. One sample was drawn upon arrival at the Emergency Department (ED) within 1 h, while the other sample was drawn within 24 h. The "comparison with the group" column indicates the statistical analysis of the percentage of IL-6 change between males and females within each group. The "Inter-group sex differences within the same sex" column compare the percentage of IL-6 change between groups of the same sex, identifying any differences in IL-6 concentrations.

References

HUNG, K.-F., HUNG, C.-H., HONG, C., CHEN, S.-C., SUN, Y.-C., WEN, J.-W., KUO, C.-H., KO, C.-H. & CHENG, C.-M. 2021. Quantitative Spectrochip-Coupled Lateral Flow Immunoassay Demonstrates Clinical Potential for Overcoming Coronavirus Disease 2019 Pandemic Screening Challenges. *Micromachines,* 12**,** 321.

LIN, S.-W., SHEN, C.-F., LIU, C.-C. & CHENG, C.-M. 2021. A Paper-Based IL-6 Test Strip Coupled With a Spectrum-Based Optical Reader for Differentiating Influenza Severity in Children. *Frontiers in Bioengineering and Biotechnology,* 9.

PAN, S.-C., WU, Y.-F., LIN, Y.-C., LIN, S.-W. & CHENG, C.-M. 2022. Paper-Based Interleukin-6 Test Strip for Early Detection of Wound Infection. *Biomedicines,* 10**,** 1585.

WANG, Y.-C., LIN, S.-W., WANG, I.-J., YANG, C.-Y., HONG, C., SUN, J.-R., FENG, P.-H., LEE, M.-H., SHEN, C.-F., LEE, Y.-T. & CHENG, C.-M. 2022. Interleukin-6 Test Strip Combined With a Spectrum-Based Optical Reader for Early Recognition of COVID-19 Patients With Risk of Respiratory Failure. *Frontiers in Bioengineering and Biotechnology,* 10.
